# Effect of Mindfulness Based Stress Reduction (MBSR) in Increasing Pain Tolerance and Improving the Mental Health of Injured Athletes

**DOI:** 10.3389/fpsyg.2018.00722

**Published:** 2018-05-15

**Authors:** Warhel Asim Mohammed, Athanasios Pappous, Dinkar Sharma

**Affiliations:** ^1^School of Sport and Exercise Sciences, University of Kent, Canterbury, United Kingdom; ^2^School of Psychology, University of Kent, Canterbury, United Kingdom

**Keywords:** injured athletes, mindfulness meditation, pain tolerance, stress, mental health

## Abstract

Literature indicates that injured athletes face both physical and psychological distress after they have been injured. In this study, a Mindfulness Based Stress Reduction (MBSR) was utilised as an intervention for use during the period of recovery with injured athletes and, to the best of our knowledge, this is the first study using MBSR as an intervention for this purpose.

**Objective:** The aim of this research was to investigate the role of MBSR practise in reducing the perception of pain and decreasing anxiety/stress, as well as increasing pain tolerance and mindfulness. An additional aim was to increase positive mood and decrease negative mood in injured athletes.

**Methods:** The participants comprised of twenty athletes (male = 14; female = 6; age range = 21–36 years) who had severe injuries, preventing their participation in sport for more than 3 months. Prior to their injury, the participants had trained regularly with their University teams and participated in official university championships. Both groups followed their normal physiotherapy treatment, but in addition, the intervention group practised mindfulness meditation for 8 weeks (one 90-min session/week). A Cold Pressor Test (CPT) was used to assess pain tolerance. In contrast, the perception of pain was measured using a Visual Analogue Scale. Other measurements used were the Mindful Attention Awareness Scale (MAAS), Depression Anxiety and Stress Scale (DASS), and Profile of Mood States (POMS).

**Results:** Our results demonstrated an increase in pain tolerance for the intervention group and an increase in mindful awareness for injured athletes. Moreover, our findings observed a promising change in positive mood for both groups. Regarding the Stress/Anxiety scores, our findings showed a notable decrease across sessions; however, no significant changes were observed in other main and interaction effects in both groups.

**Conclusion:** Injured athletes can benefit from using mindfulness as part of the sport rehabilitation process to increase their pain tolerance and awareness. Further research is required to assess whether increasing pain tolerance could help in the therapeutic process.

## Introduction

Sport injuries are a considerable public health concern. The impact of the injured athlete extends beyond the individual. Although it may impact on their seasonal and potential career performance, it additionally impacts upon the clubs and organisations for whom they perform. Furthermore, it leads to a greater general burden on the health service. Every year, in the United Kingdom, there are 29.7 million injuries among athletes (Nicholl et al., 1995). This is in line with studies by Nicholl et al. (1995) and Leppänen et al. (2014), who indicate that, although there are many health benefits from participating in sport activities, risks should be expected. This occurs when athletes become injured and are out of their sports for a considerable period, or when a termination of a player’s career occurs because of severe re-occurring injuries. Heaney (2006) and Reese et al. (2012) report that injuries can affect athletes’ mental health by triggering depression and anxiety, decreased self-esteem, loss of identity, anger, isolation, fear, and tension. It is worth noting, that sport injuries include both psychological and physiological effects on athletes (Ruddock-Hudson et al., 2014). Peterson and Renstrom (2000) clarify that sport injuries are caused by trauma. They divide sport injuries into different levels of trauma: injuries that are caused by overuse syndromes and those caused by traumatic injuries. Overuse syndromes are common among athletes because of the duration of training and highly intense exercise, whereas traumatic injuries occur because of the impact of a large force, often resulting in a high level of pain.

Furthermore, sport injuries lead to an imbalance and discomfort in life for athletes after injury and this physical inability prevents them from achieving in their sport (Reese et al., 2012). From this perspective, recent evidence discussed by Ford et al. (2000), Tracey (2003), Heaney (2006), Vergeer (2006), Reese et al. (2012), Arvinen-Barrow et al. (2014), Ruddock-Hudson et al. (2014), and Tatsumi and Takenouchi (2014) suggests that psychological interventions are important for athletes to play an effective role in the rehabilitation process after being injured. Psychological interventions can lead to a reduction in negative thoughts and moods. Heaney (2006) reveals that there are many studies that have been undertaken using psychological interventions to enhance athletes’ attitudes and reduce negative thoughts as a strategy of the injury rehabilitation process (Ford and Gordon, 1998; Francis et al., 2000; Hemmings and Povey, 2002). Crossman (1997) and Dawes and Roach (1997) state that it is common, during and after injury, for athletes to have negative thoughts and experiences. Therefore, understanding the psychological response is the first step in organising rehabilitation for injured athletes, because emotions stimulate tension and worry (Crossman, 1997). In other words, both tension and worry impede athletes from achieving their optimum performance and hinder the injury rehabilitation process. In support, Ivarsson et al. (2013) also emphasise that in recent years, theoretical concepts, empirical studies, and applied knowledge in the psychology of injury are widely used as part of the rehabilitation process of injured athletes.

Sport injuries can affect injured athletes’ teammates, coaches, and family members. Therefore, their social support can have a positive effect on athletes and help them to return to their sports (Crossman, 1997). Regarding the social factor in sport rehabilitation, Rees et al. (2010) emphasise that receiving support from family, medical staff, coaches, and friends can enable athletes to cope with psychological distress. In this vein, Podlog et al. (2014) stress that the duration of the sport rehabilitation process could be shorter, for injured athletes, if they continue to connect and socialise with teammates, keep their fitness level high, have a love of sports, and reach their personal goals. Calvert (2015) also stressed that utilising psychological interventions with athletes is useful during the rehabilitation process. The reason is that athletes’ beliefs, emotions, and thoughts influence the way their body responds after injury. More specifically, there is an interaction between body and mind and this interaction can be utilised for two purposes. The first is to support injured athletes in the rehabilitation process. The second is that injured athletes become more confident in avoiding the risk of injuries (Calvert, 2015).

With respect to the role of mindfulness meditation in current research, Arvinen-Barrow and Walker (2013) mention that as a part of sport injury rehabilitation, mindfulness can be an effective instrument to achieve a relaxed state of body and mind. Moreover, it can enable an individual to gain more awareness and acceptance about their situation as an injured athlete (Arvinen-Barrow and Walker, 2013). Besides, it might be suitable to turn their attention to psychotherapy treatment, seeking to confirm the correct course of action for rehabilitation (Arvinen-Barrow and Walker, 2013). Venkatesh et al. (1997) report that practising meditation, in the long-term, leads to considerable changes in awareness. Furthermore, the study has investigated significant changes in self-awareness, arousal, and perceptual experience. Stahl and Goldstein (2010) emphasise being in the present moment, or living in the body, by paying attention to, and being conscious of, physical sensations. Therefore, a ‘body scan’ is a very convenient mode with which to connect with one’s mind and body. In this way, a body scan can be an effective technique for the reduction of physical pain, anxiety and stress. More importantly, it has been reported that mindfulness meditation is beneficial for healing those suffering from pain. In addition, Ivarsson (2015) refers to elite football players who undertook psychological interventions based on attention, and they were able to diminish sports injuries. He also recommends daily mindfulness exercises to lessen the risk of injury.

In this vein, mindfulness-based intervention has also flowed into sport performance. Literature has shown beneficial consequences in improving athletes’ performance. Birrer et al. (2012) emphasise that cultivating mindfulness practise with athletes can be taught via a different mechanism. For instance, mindful breathing could be introduced as a ‘non-sports setting’; however, it can be also being incorporated into athletes’ sport attitude by accepting and not judging those attitudes. In other words, mindfulness practise can encourage athletes to accept whatever situations they might face during involvement with their sports, and not judge them. They also demonstrate the capability of incorporating mindfulness practise into sport training. Another example of incorporating mindfulness practise in sports, as mentioned by Birrer et al. (2012), was a body-scan, that could take place at the end of a sport training programme or during the cool-down process.

By the same token, numerous empirical trials are analysed by Sappington and Longshore (2015). They discuss promising evidence of the usefulness of mindful practise in sport performance. As such, Thompson et al. (2011) suggest applying a long-term mindfulness approach called Mindful Sport Performance Enhancement (MSPE), as a promising intervention that can enhance athletes’ performance.

Therefore, this study aims to use a common meditation technique, based on Mindfulness-Based Stress Reduction (MBSR), as an intervention for utilisation during the recovery period of injured athletes. The aim of this research was to investigate the role of MBSR practise in reducing the perception of pain and anxiety/stress and increasing pain tolerance and mindfulness. Additionally, the aim was to increase positive mood and decrease negative mood in injured athletes.

Our hypothesis was that practising regular mindfulness will increase pain tolerance, and awareness, in injured athletes. Furthermore, it will reduce the perception of pain and decrease negative mood in their daily lives.

## Materials and Methods

### Participants

A total of 20 injured athletes, who were all university students, were recruited; Flyers were placed in different university locations such as academic departments, clinics, student unions, and exercise facilities, emails were sent to all students through the students’ support officer at the School of Sport and Exercise Sciences, and participants were recruited through word-of-mouth and by asking therapists to refer injured athletes.

Regarding the inclusion criteria, participants who were involved in this intervention were athletes who trained regularly with their teams in university. They participated in official university championships but were not elite athletes.

In addition, participants in both groups had been away from their sports due to injury for 3–6 months. The participants (injured athletes) were from various kinds of sports as well as typology of injury. Both males and females could take part in this intervention. The age of participants was between 18 and 45 years. Participants who took part in the intervention group were asked to attend all sessions of the MBSR. Similarly, they were asked to complete all assessment tools and CPT during the period of the MBSR programme. As such, the same procedure was followed in the control group prior to and after each physiotherapy session and during the MBSR programme. Furthermore, they were asked to read the participant information sheet carefully, which included all the instructions, before they signed the informed consent form to take part in the study.

In relation to the exclusion criteria, participants who self-reported having diabetes (Type I or II), haemophilia, Reynaud’s syndrome, fainting, seizures, any recent cuts to the hand, or cardiovascular disorders were excluded from the study. Any absence from any mindfulness session in the intervention group or physiotherapy session for the control group, meant that the participants were excluded from the programme. Participants who withdrew from the MBSR programme were excluded from this study.

The randomisation process was designed to approach 20 injured athletes in both the intervention and control groups. The first participant who visited the sport clinic and signed the informed consent was allocated to the intervention group. Likewise, the second participant who visited the sport clinic was assigned to the control group. Pairs of participants, as they arrived at the sport clinic at the same time, were assigned randomly by a third person (blind to the aims of the study) to one of the two groups. Four participants dropped out of the study, two dropped out after signing the consent form. Another two withdrew after starting the MBSR but were replaced.

Demographic information for each participant that completed the study is presented in **Table 1**. The two groups did not differ in age, *t*(18) = 0.083, *P* = 0.935, (*M* intervention = 28.9 years, *SD* = 6.21; *M* control = 28.7, *SD* = 4.47) or gender, *χ*^2^ = 0.952, *P* = 0.329. All participants received physiotherapy treatment at a sports therapy clinic when they had been away from their sports for three to 6 months.

**Table 1:** Demographic details for each participant in each of the groups in terms of age, gender, clinical characteristics of the injury and sporting activities.

Age	Gender	Typology of Injury	Physical Activity	Side of Injury	Side of Body Tested for CPT
**Intervention Group**				
36	Male	Ankle injury	Tennis	Right Ankle	Right Hand
36	Male	Wrist injury	Kickboxing	Right Wrist	Right Hand
34	Male	Knee injury	Bodybuilding	Right Knee	Right Hand
32	Female	Hips Injury	Running	Left Side	Left Hand
32	Male	Low Back pain	Running	Low Back pain	Right Hand
31	Male	Shoulder injury	Football	Left Shoulder	Right Hand
24	Male	Shin injury	Basketball	Right Side	Right Hand
22	Male	Collateral-Ligament	Basketball	Right Knee	Right Hand
21	Male	Knee ACL	Running	Right Knee	Right Hand
21	Female	Knee ACL	Basketball	Right Knee	Right Hand
**Control Group**				
36	Male	Knee (ACL)	Football	Right Knee	Right Hand
33	Male	Low Back pain	Kickboxing	Low Back Pain	Right Hand
31	Male	Arm injury Knee injury	Running	Left Arm	Right Hand
31	Female		Cyclist	Right Knee	Right Hand
30	Male	Peroneal tendon subluxation	Cyclist	Left Side	Right Hand
29	Male	Shoulder injury	Bodybuilding	Left Side	Right Hand
26	Female	Elbow injury	Basketball	Left Elbow	Right Hand
26	Male	Ankle	Cyclist	Right Ankle	Right Hand
24	Female	Knee (ACL)	Running	Right Knee	Right Hand
21	Female	Big Toe/ Proximal phalanx	Running	Left Side Toe	Right Hand

*ACL, Anterior cruciate ligament. The labels used in the typology of injury were labels given by the injured athlete. These injuries either occurred during participation in the sport or during training. The two groups did not differ in age, t(18) = 0.083, P = 0.935, (m intervention = 28.9 years, SD = 6.21; m control = 28.7, SD = 4.47) or gender, χ^2^ = 0.952, P = 0.329.*

### Procedure

Ethical approval for the study was obtained from the Ethics Committee, School of Sport and Exercise Sciences.

All the participants gave informed consent prior to starting the study. All the participants saw the participant’s information sheet (PIS) and signed the consent form. The PIS contained information regarding the procedure involved in this study, such as the purpose of the study, what kind of population could take part, whether there were any benefits and risks involved in taking part, confidential issues, and contact details.

In week zero (week_0) and nine (week_9) of the study, all the participants completed the cold pressor test (CPT). During weeks one to eight, participants in the intervention group completed three questionnaires (MAAS, DASS, and POMS) before and after each formal meditation session. Injured athletes in the control group who did not receive MBSR were also asked to complete the CPT in week zero and week nine. Regarding the quantitative measurements, they completed all the questionnaires before starting their clinical session and at the end of the treatment.

The intervention in this study was based on the original version of the MBSR, which was developed by Kabat-Zinn 1979 at the University of Massachusetts Medical Centre in Worcester (Kabat-Zinn, 2013). Notably, MBSR consists of 8 weeks of coursework lasting about 2.5 h per week in a group session. In this study, the procedure was modified due to the nature of the severely injured athletes’ state. Hence, carrying out meditation for 2.5 h was not medically reasonable because the physically injured athletes were not capable of practising meditation for that duration, due to suffering pain whilst maintaining a stable body posture (see **Table 2**).

**Table 2:** Mindfulness-Based Stress Reduction (MBSR) that was used in this study with injured athletes in the intervention group.

Weeks	Formal meditation practise (90 min)	Informal meditation practise (20 min)
Week 0	– Participant information sheet – Consent forms – Cold Pressor Test (CPT)	Participants given the CD guide of MBSR programme to practise at home.
Week 1 - Week 8	– Participants were asked to complete three questionnaires (MAAS, DASS and POMS), approximately 15 min. – 10–15 min of mindful check-in and sharing ideas about mindfulness meditation practise. – 30 min formal meditation practise with researcher. The formal session included these meditation skills (sitting/laying down meditation, mindful breathing and body scan meditation). – Participants were asked to complete three questionnaires (MAAS, DASS, and POMS) for the second time at the end of the meditation session. – 10–15 min of mindful check-in and sharing of ideas about mindfulness meditation practise. – Participants attended the lab on the same day each week.	– 20 min daily meditation practise. The CD guide of MBSR programme includes (sitting/laying down meditation, mindful breathing, body scan meditation, mindful eating, mindful walking meditation, meditation for anxiety and stress, mindful lying yoga, mindful standing, yoga, and loving kindness meditation. – Participants were free to choose the skills they would apply or listen to.
Week 9	Participants repeated the Cold Pressor Test (CPT) at the end of the MBSR.	

Likewise, gathering all injured athletes in a group session, at the same time, was not possible because of their physiotherapy treatments and availability. Therefore, individual sessions were run with each athlete.

All participants in the study followed their normal physiotherapy treatment. Notably, for the duration of the sport therapy, each injured athlete followed therapy advice specific to their injury.

Participants were asked to come to a specific room, which was adapted to run the mindfulness practise in a noiseless and unobtrusive space at the School of Sport and Exercise Science. The participants started each session by completing three types of questionnaire that lasted approximately 15 min. They then spent 10–15 min on a mindful check-in and shared ideas about mindfulness meditation. After 30 min of meditation (mindful breathing, body scan meditation, and sitting meditation) the same questionnaires were completed, followed by the sharing of ideas about meditation and their body sensations. Consequently, the participants spent about 90 min in each session with the first author.

In addition to 90 min of formal meditation practise with an instructor and as a part of the MBSR programme, each participant was given a CD guide of meditation practise to listen to and were asked to practise at home for between 20 and 30 min per day. The informal meditation practise in this study was based on the CD guide that was delivered to injured athletes to apply at any time during their daily activities.

In this study, the CD from the ‘mindfulness-based stress reduction workbook’ by Stahl and Goldstein (2010) was offered to injured athletes and at the end of the MBSR they were returned to the researchers. It is important to note that, many researchers have followed different levels of practise of MBSR in their research, such as work by Mackenzie et al. (2006) and Bergen-Cico et al. (2013). It should also be noted that injured athletes in both groups received physiotherapy treatment according to their specific injury.

### Cold Pressor Test

As per previous studies, injured athletes started the CPT test by sitting down and submerging one hand (participants chose their preferred hand) in a bucket of cold water between 0 and 2°C for a maximum of 8 min (Angius et al., 2015). They were instructed to put their hand in the ice bucket and keep it in for as long as possible but for no longer than 8 min. All participants placed their hand in the ice bucket so that the whole hand was submerged up to the level of the wrist.

The pain tolerance measure was described as the time between submersion and removal of the hand from the cold water. CPT is appropriate for this research as it is safe, time efficient, and is a reliable method widely utilised to measure pain (Mitchell et al., 2004; Wirch et al., 2006; Angius et al., 2015).

### Visual Analogue Scale (VAS)

Injured athletes recorded a mark on a 10 cm straight line, anchored with the labels *no pain* and *most pain* at each end, to indicate the degree of pain experienced when removing their hand from the water in the CPT. VAS is a popular and reliable assessment tool for measuring pain (Johnson, 2005). The interclass correlation between the two sessions was *r*(12) = 0.807.

### Mindful Attention Awareness Scale (MAAS) (Brown and Ryan, 2003)

This is a 15-item questionnaire that measures the frequency of mindful states in everyday life, using general and situation-specific statements. Responses are given on a six-point scale, from one *(almost always)* to six *(almost never)* with higher scores representing greater mindfulness. The objective of using the MAAS scale is to obtain the level of participants’ mindful awareness across the 8 weeks of the study. Across the two sessions and 8 weeks, Cronbach’s α ranged between 0.946 and 0.838.

### Depression Anxiety and Stress Scale (DASS) (Lovibond and Lovibond, 1995)

The DASS was used to assess the level of anxiety and stress during the 8 weeks of the study. The DASS scale contains 42 items, consisting of three subscales to evaluate depression, anxiety, and stress. Only the anxiety and stress section of the scale were administered, with participants reporting the symptoms they were currently experiencing. The anxiety scale comprises four factors: skeletal muscle effects, the subjective experience of the effect of anxiety, situational anxiety, and autonomic arousal. In contrast, the stress scale assesses nervous arousal, irritability/being over-reactive and impatient, being upset/agitated, and having difficulty relaxing. The rating scale is divided between zero *(did not apply to me at all)* to three *(applied to me very much, or most of the time)* (Lovibond and Lovibond, 1995). Across the two sessions and 8 weeks, Cronbach’s α varied between 0.941 and 0.436.

### Profile of Mood States (POMS) (Terry et al., 2003)

Injured athletes completed the POMS prior to and after each session during the 8-week MBSR programme. Participants answered the POMS according to how they felt at the time and they chose from a rating scale of zero *(not at all)* to four *(extremely*) (Terry et al., 2003). Across the two sessions and 8 weeks, Cronbach’s α varied between 0.843 and 0.525.

### Statistics Analysis

All data was calculated as means and Standard Error to assess the pre- and post-meditation practise during each week and for both the intervention and control group.

Pain perception scores from the VAS and pain tolerance were analysed using a two-way mixed analysis of variance, with Group (intervention, control) as the ‘between’ subject factor and Time (week_0, week_9) as the ‘within’ subject factor. Scores from each of the questionnaires (MAAS, DASS, and POMS) were analysed using a three-factorial mixed analysis of variance with Group (intervention, control) as the ‘between’ subject factor and Time (weeks 1–8) and Session (pre and post) as the ‘within’ subject factors (see **Tables 3**, **4**).

**Table 3:** Mean, standard errors, and partial eta squared (PES) for the main effect of session for the POMS, MAAS and DASS questionnaires for the intervention group and control group.

Dependent Variables		Session			
		Pre	Post	*P*	*PES*
POMS: depression-dejection	Mean	2.76	1.71	0.001	0.49
	Standard error	0.47	0.28		
POMS: tension-anxiety	Mean	3.94	2.58	<0.001	0.545
	Standard error	0.51	0.39		
POMS: anger-hostility	Mean	2.75	2.22	0.165	0.104
	Standard error	0.54	0.43		
POMS: vigour-activity	Mean	6.46	6.65	0.617	0.014
	Standard error	0.76	0.81		
POMS: fatigue-inertia	Mean	3.74	2.49	0.004	0.385
	Standard error	0.51	0.42		
POMS: confusion-bewilderment	Mean	2.74	1.75	<0.001	0.294
	Standard error	0.43	0.27		
MAAS	Mean	61.26	64.81	0.001	0.478
	Standard error	2.49	2.56		
DASS-anxiety	Mean	6.18	4.87	0.008	0.328
	Standard error	1.00	0.82		
DASS-stress	Mean	9.96	7.74	0.001	0.471
	Standard error	1.13	1.11		

**Table 4:** Mean and standard errors and partial eta squared (PES) for the main effect of Time for the POMS, MAAS and DASS questionnaires for the intervention group and control group.

Dependent Variables		Time									
		1	2	3	4	5	6	7	8	*P*	PSE
POMS: depression-dejection	Mean	3.35	3.01	1.95	2.17	1.97	2.17	1.87	1.38	0.004	0.152
	Standard error	0.61	0.64	0.39	0.47	0.37	0.52	0.41	0.38		
POMS: tension-anxiety	Mean	4.63	4.68	2.62	2.66	2.74	3.83	2.49	2.40	<0.001	0.248
	Standard error	0.74	0.66	0.41	0.50	0.55	0.71	0.47	0.52		
POMS: anger-hostility	Mean	3.12	3.46	2.17	2.16	2.11	2.59	2.27	2.00	0.165	0.105
	Standard error	0.61	0.80	0.46	0.53	0.39	0.50	0.54	0.51		
POMS: vigour-activity	Mean	6.87	6.63	6.63	6.62	6.17	6.42	6.65	6.46	0.617	0.03
	Standard error	0.69	0.75	0.93	0.86	0.67	0.89	0.95	1.02		
POMS: fatigue-inertia	Mean	5.11	4.59	2.48	3.50	2.76	2.26	1.99	2.21	0.004	0.327
	Standard error	0.70	0.77	0.43	0.76	0.48	0.52	0.44	0.47		
POMS: confusion-bewilderment	Mean	3.56	3.15	2.08	2.00	2.46	2.03	1.48	1.22	0.001	0.262
	Standard error	0.57	0.54	0.30	0.40	0.47	0.45	0.31	0.29		
MAAS	Mean	57.90	58.15	62.70	64.48	63.40	63.00	66.20	68.48	0.001	0.264
	Standard error	2.58	2.78	2.64	2.84	2.65	2.93	3.04	3.09		
DASS-anxiety	Mean	6.20	6.58	5.35	5.90	5.35	5.15	5.10	4.55	0.611	0.041
	Standard error	0.94	0.87	1.21	1.34	1.23	1.47	1.04	0.72		
DASS-stress	Mean	9.52	10.97	10.33	8.73	9.50	8.25	7.18	6.33	0.001	0.166
	Standard error	1.26	1.47	1.38	1.46	1.04	1.28	1.45	1.26		

## Results

### Analysis of Pain Perception and Pain Tolerance

**FIGURE 1:**
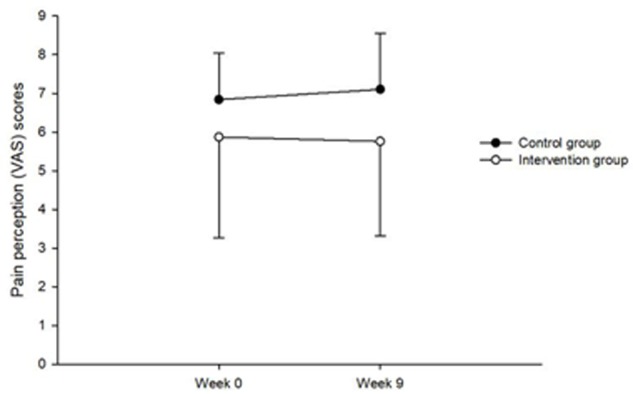
Showing the Time × Group interaction for pain perception.

**FIGURE 2:**
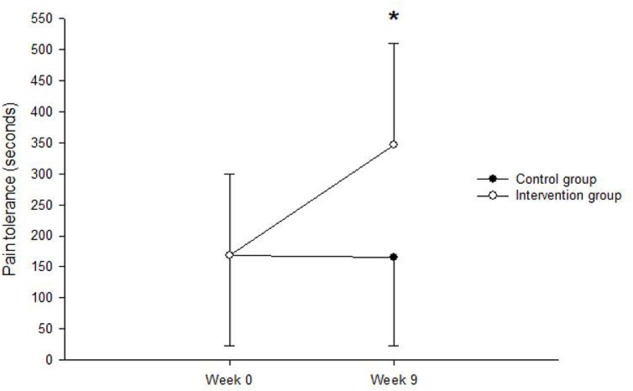
Showing the Time × Group interaction for pain tolerance.

Analysis of pain perception scores showed no significant main or interaction effects (all *F’s* < 0.36, *P’s* > 0.18, partial eta squared (PES) < 0.1, see **Figure 1**. Analysis of pain tolerance scores indicated a significant main effect of Time, *F*(1,18) = 12.21, *P* = 0.003, PSE = 0.4 but no main effect of Group *F*(1,18) = 2.29, *P* = 0.148. However, there was a Time × Group interaction, *F*(1,18) = 13.12, *P* = 0.002, PES = 0.422. **Figure 2** indicates that the increases were only in the intervention group. Further analysis showed no significant difference between the two groups at week_0, [*t*(18) = 0.006, *P* = 0.9], but a significant difference at week_9 [*t*(18) = 2.66, *P* = 0.016].

### Mindful Attention Awareness Scale

The MAAS scores increased over the two Sessions, *F*(1,18) = 16.45, *P* = 0.001, PSE = 0.478 and over the 8 weeks, *F*(7,126) = 6.45, *P* < 0.001, PSE = 0.264. There was also a main effect of Group, *F*(1,18) = 5.34, *P* = 0.033, PSE = 0.229), indicating higher MAAS scores for the intervention group (*m* = 68.79, *s* = 3.52) than the control group (*m* = 57.29, *s* = 3.52). There was a greater change in the MAAS scores across the Session for the intervention group (pre = 66.16, post = 71.41) than for the control group (pre = 56.36, post = 58.21), the Group *x* Session interaction, *F*(1,18) = 3.77, *P* = 0.034 (one-tailed), PSE = 0.173 was significant.

### Profile of Mood States

These results indicated that there were general changes in mood across Session and Time for depression, tension, fatigue and confusion scores. All other main and interaction effects were not significant (*P* > 0.06). The main effects of Session and Time are illustrated in **Tables 3**, **4** respectively, for each measure of mood.

### Depression Anxiety and Stress Scale

Anxiety scores indicated a notable decrease in anxiety across Sessions, *F*(1,18) = 8.80, *P* = 0.008, PSE = 0.328 see **Table 3**. All other main and interaction effects were not significant (*P* > 0.2). Stress scores indicated a decrease across Sessions, *F*(1,18) = 16.04, *P* = 0.001, PSE = 0.471 and Time *F*(7,126) = 3.59, *P* = 0.001, see **Tables 3**, **4**. All other main and interaction effects were not significant (*P* > 0.06).

## Discussion

The objective of this study was to investigate whether MBSR has any effect on reducing pain and the improvement of the mental health of injured athletes during an 8-week programme. Results showed that there was an increase in pain tolerance, and therefore less sensitivity to pain, in the intervention group. This study suggested that MBSR could be used by injured athletes to manage their pain. Hence, meditation practise might provide them with an ability to manage their injury. It was also observed that injured athletes who had taken part in the intervention group gained beneficially from MBSR as an additional tool during the sport rehabilitation process. Self-regulation practise could also improve pain management through attitudes that emerged from MBSR. Consequently, pain tolerance increased further in injured athletes, who received MBSR, compared to their peers in the control group. To support this view, Kabat-Zinn (2013) states that ‘keeping particular attitudes in mind is actually part of the training itself, a way of directing and channelling your energies so that practitioners can be most effectively brought to bear in the work of growing and healing’ (p. 21).

This supports previous research, which found that people who participated in 8 weeks of a Mindfulness Based Stress Reduction experience a significant reduction in their pain, compared to the control group that followed a health education protocol (Morone et al., 2009). Additionally, Lykins and Baer (2009) and Nehra et al. (2012) indicate the self-regulation benefits of MBSR on pain management and well-being. Essentially, Baer (2003) refers to the research that has been conducted on the influence of MBSR on patients with pain disorders. It included four studies that were the same as those of Kabat-Zinn et al. (1985, 1987), and Radolph et al. (1999). All this research collected data about chronic pain with patients who applied MBSR. In addition, the results of these studies showed that there was a significant enhancement in pain grades for patients. Furthermore, Zeidan et al. (2010) mention that 3 days of a brief mindfulness intervention is effective in diminishing pain and increasing psychological status. Our results highlight that MBSR can be used in a group of injured athletes to increase pain tolerance.

However, the VAS scores in perception of pain showed that there was no difference in the intervention or control groups pre- and post-programme. Based on the instructions of CPT, injured athletes in both groups could take their hands out of the water when they experienced pain. This might predicate their similar levels of perception of pain.

The MAAS results showed that mindful awareness was higher immediately after 90 min of MBSR. This change was more significant in the intervention group than in the control group. In other words, participants in the intervention group understood how to pay attention, live in the present moment, and increase their level of body awareness, without criticising themselves through specific instructions, which they received from MBSR. That could be the main reason for the higher scores of MAAS in the intervention group.

It was also observed that mindful awareness increased across the 8 weeks. As this was true of both groups, this is probably due to both groups receiving physiotherapy treatment. Physiotherapy relies on touch to treat patients; therefore, our expectation was that it would lead to an improvement in their mindful awareness, possibly due to the focus of attention on a specific part of the body, after 8 weeks of treatment. The MAAS results in this study support previous research, which has shown an increase in mindfulness skills over time with cancer patients (Brown and Ryan, 2003). Correspondingly, athletes who contributed to research by Goodman et al. (2014), were taken from 26 colleges and several experimental groups comprising of eight athletes, whereas, the control group consisted of 13 male athletes from sport teams. As a result, there were significant results in the experimental group, with greater attention to their goals regarding mindful exercise than student athletes from the control group. Another possible explanation for our findings is that by practising mindfulness meditation on a regular basis, injured athletes improve the regulation of their emotions. To support this interpretation, Siegel (2008) stated that mindfulness application leads to combatting emotional dysfunction, a reduction in negative attitudes, a capability to regulate emotion, and an improvement in patterns of thinking. Moreover, a study by Azulay and Mott (2016) found encouraging results in relation to mindfulness meditation practise with a mixed brain injury. They also observed that awareness had increased in patients with a stroke condition, as they were more mindful about their disability at the end of the treatment. They explained their findings were a consequence of regular meditation, providing patients with the ability to manage their pain from physical injuries.

A control group that did not receive physiotherapy treatment would be distinguished from any natural increase in mindfulness with time. In the control group, athletes could have directed their attention to their injury, which might have been sustained throughout the 8 weeks. Alternatively, it could be that the injury increases worry or rumination of the consequences of the injury. Then, as the injury heals over time, these decrease, subsequently improving mindfulness awareness.

Regarding mood changes, a consistent pattern emerged over Time and over Sessions. We found a general decrease in mood for depression-dejection, tension-anxiety, fatigue-inertia, and confusion-bewilderment. There were no significant changes for anger-hostility or vigour-activity. As these main effects did not interact with the group, this suggests no additional benefit was gained from the MBSR. It is important to note that there was mood decrease in the control group. This could be due to the physiotherapy though, again, a control group (without physiotherapy) would be needed to answer this question. It could be that as the injury improves over time (either due to physiotherapy or not) mood decreases. The lack of any change in tension-anxiety and vigour-activity may be because injured athletes were not engaged with their physical activities and therefore, they did not feel active enough during those 8 weeks.

In relation to stress and anxiety scores, the results showed a notable decrease across sessions, however; no significant changes were observed in the other main interaction effects, although, a sizable body of literature found a positive influence of MBSR in reducing anxiety and stress (Ott et al., 2006; Pradhan et al., 2007; Keng et al., 2011). A potential explanation for this might be the injured athlete’s state of mind at the time of completing the DASS questionnaires. Furthermore, the last therapeutic process might also have an effect on injured athletes’ scores.

According to the results of this study, injured athletes can benefit from using mindfulness meditation as a part of the sport rehabilitation process to increase their pain tolerance and the level of mindful awareness.

### Implications

The main aim of this study was to explore the usefulness of MBSR in a sport injury rehabilitation context. Based on the results of this study, incorporating the MBSR programme into sport therapy helped injured athletes to increase their pain tolerance as well as mindfulness, and had a positive effect on their recovery from an injury. This study suggests that there is considerable scope for including some formal mindfulness components into the professional training of sports injury rehabilitation professionals. More specifically, regarding the significant mental nature of pain, mindfulness can become an essential part in the therapeutic toolkit of sport therapy.

This is consistent with Pen and Fisher (1994), who suggest that the ability of an injured athlete to support pain is related to how quickly the athlete recovers from an injury. However, regarding therapeutic duration, further research is needed to understand whether MBSR could support injured athletes during the recovery period.

### Limitations of This Study

• The effectiveness of MBSR on the participants’ injury-related pain was not measured. In this study there were different types and extents of sport injuries, making it difficult to compare different categories of injury.
• In this study the procedure was modified due to the nature of the injured athletes’ state after suffering severe injuries. Therefore, individual sessions were run with each athlete. As such, the time of the formal meditation sessions was one 90-min session per week.
• The sample size was a limitation for the study; only 20 injured athletes completed the study. This was partially due to the difficulty in recruiting injured athletes. Further research is therefore required to substantiate and generalise the findings of this study.
• The gender and the typology of sports injuries were other limitations of this study that should be taken into the consideration for future research.
• An additional active control group could be beneficial in future research.
• Another potential limitation to this study was the assessment of informal meditation practise. Injured athletes were asked to complete numerous requirements during the MBSR programme, thus affecting their participation in this study.

## Author Contributions

WM collected the data, conducted the preliminary statistical analyses, and wrote the first draught of the manuscript. AP generated the original idea for the study and was responsible for interpreting the data. DS led the design of the methodology and contributed to the data processing. All authors contributed to the interpretation of the data, reviewed/edited the manuscript, and approved the final version.

## Conflict of Interest Statement

The authors declare that the research was conducted in the absence of any commercial or financial relationships that could be construed as a potential conflict of interest.
